# *Lactobacillus gasseri* SBT2055 suppresses fatty acid release through enlargement of fat emulsion size *in vitro* and promotes fecal fat excretion in healthy Japanese subjects

**DOI:** 10.1186/s12944-015-0019-0

**Published:** 2015-03-20

**Authors:** Akihiro Ogawa, Toshiya Kobayashi, Fumihiko Sakai, Yukio Kadooka, Yoshihiro Kawasaki

**Affiliations:** Milk Science Research Institute, Megmilk Snow Brand Co. Ltd., 1-1-2 Minamidai, Kawagoe, Saitama 350-1165 Japan; Public Relations Department, Megmilk Snow Brand Co. Ltd., 13 Honshiocho, Shinjuku-ku, Tokyo 160-0003 Japan

**Keywords:** Probiotics, *Lactobacillus gasseri* SBT2055, Lipid absorption, Lipase, Fat emulsion, Droplet size, Fecal fat excretion

## Abstract

**Background:**

*Lactobacillus gasseri* SBT2055 (LG2055) has been shown to prevent abdominal adiposity, and suppression of lipid absorption is considered a possible mechanism, detail of which, however, are poorly understood. In the present study, we evaluated the effects of LG2055 on fat hydrolysis by determining pancreatic lipase activity and fat emulsion properties *in vitro*. We also examined whether LG2055 influences fecal fat excretion in humans.

**Methods:**

Pancreatic lipase activity was investigated *in vitro* using an artificially prepared fat emulsion and 4-methylumbelliferyl oleate (4-MUO) as substrates. The concentrations of free fatty acids and 4-methylumbelliferone were quantified. Fat emulsion droplet size was measured using a particle size analyzer. The clinical study was performed as a double-blind, randomized, placebo-controlled trial. Subjects consumed 100 g of fermented milk (FM)/d, either with or without LG2055 supplementation, for seven days. Fecal samples were collected during three-day pre-observational and FM intake periods and fecal fat levels were determined.

**Results:**

LG2055 dose-dependently suppressed lipase activity in the fat emulsion assay but not in the 4-MUO assay. LG2055 dose-dependently increased fat emulsion droplet size. The effects of LG2055 on lipase activity and fat emulsion properties were increased compared with four other tested strains (*Lactobacillus gasseri* SBT0317, *Lactobacillus gasseri* JCM1131^T^, *Lactobacillus. delbrueckii* subsp. *bulgaricus* JCM1002^T^ and *Streptococcus thermophilus* ATCC19258^T^). In our clinical study, fecal fat level after FM intake was significantly increased compared with that observed before FM intake in the LG2055-containing active FM group but not the control FM group lacking LG2055.

**Conclusions:**

LG2055 increased fat emulsion droplet size, resulting in the suppression of lipase-mediated fat hydrolysis. The influence of LG2055 on the physicochemical properties of fat emulsion provides a mechanism for the probiotic-mediated suppression of lipid absorption and promotion of fecal fat excretion in humans.

**Trial registration:**

UMIN000015772

## Background

*Lactobacillus gasseri* SBT2055 (LG2055), a probiotic lactic acid bacterium originating in the human intestine [1,2], has an ability to improve the intestinal environment [3] and exerts anti-obesity effects in rats [4-6] and mice [7]. Our previous clinical studies in Japanese adults with overweight exhibited a significantly decreased visceral fat area, body weight, body mass index (BMI), and waist and hip circumferences following consumption of fermented milk containing LG2055 at 200 g/d for 12 weeks [8,9].

Suppression of lipid absorption in the small intestine has been proposed as a potential mechanism for the anti-obesity effects of LG2055. Hamad *et al.* evaluated lymphatic lipid content in rats with permanent cannulation of the thoracic duct [4]. They demonstrated that rats fed a diet containing fermented skim milk supplemented with LG2055 showed a lower maximal rate of lymphatic lipid absorption compared with rats fed a diet containing non-fermented skim milk; these findings were supported by the observation of increased fecal fatty acid excretion [4]. Furthermore, our recent study showed that Japanese hypertriacylglycerolemic subjects who consumed fermented milk containing LG2055 at 200 g/d for 4 weeks demonstrated significantly decreased postprandial serum lipid concentrations after the intake of oral fat-loading test meals [10]. Serum lipid concentrations are influenced by not only lipid absorption via diet but also lipid metabolism. Therefore, estimation of fecal lipid excretion is a more direct index to examine dietary lipid absorption.

Dietary lipid digestion undergoes several complex processes prior to mucosal absorption in the small intestine. Pancreatic lipase is a key enzyme for lipid absorption because the majority of lipolysis is carried out in the duodenum by pancreatic lipase that is secreted from the pancreas and hydrolyzes dietary lipid-derived triacylglycerol into glycerol and fatty acids [11]. Dietary triacylglycerol, the major source of dietary fat, is not directly absorbed in the intestine unless it has been hydrolyzed by pancreatic lipase. Therefore, suppression of lipase-mediated fat hydrolysis is an effective approach for suppression of dietary triacylglycerol absorption [12].

Suppression of lipase-mediated fat hydrolysis is mainly associated with two mechanisms: the first is direct enzymatic inhibition by an inhibitor like orlistat, which is a potent pancreatic lipase inhibitor used as a pharmaceutical agent for the management of obesity [13]; the second is associated with modification of fat emulsion properties. Fat emulsion interface properties, including droplet size and specific surface area, influence the effects of lipase-mediated fat hydrolysis on dietary fat absorption [14]. As fat emulsion particle diameter increases, specific surface area decreases. Thus, lipase-mediated fat hydrolysis is sensitive to fat emulsion size [15], and the change in fat emulsion droplet size is critical for modification of fat digestion and absorption. However, the mechanism of LG2055-mediated suppression of lipid absorption is unclear.

In this study, we investigated the mechanism associated with suppression of lipid absorption by the probiotic bacterium LG2055. We determined LG2055 effects on pancreatic lipase-mediated hydrolysis of an artificial fat emulsion or synthetic substrate (lipase activity) and measured fat emulsion droplet size *in vitro* using a simple oil-in-water emulsion as a physiological model. We also examined whether intake of LG2055 altered fecal fat excretion in healthy Japanese subjects.

## Methods

### *In vitro* study

#### Materials

Triolein, taurocholic acid, pancreatic lipase (type VI-S, from porcine pancreas), orlistat, and 4-methylumbelliferyl oleate (4-MUO) were purchased from Sigma-Aldrich Co., Ltd. (St. Louis, MO, USA). *N*-Tris (hydroxymethyl) methyl-2-aminoethanesulfonic acid (TES) was purchased from Dojindo Laboratories (Kumamoto, Japan). Lecithin from egg, NEFA C Test Wako, and catechin mixture from green tea (product number 032–18231) were purchased from Wako Pure Chemical Industries Co., Ltd. (Osaka, Japan).

#### Preparation of LG2055 and other bacteria

*Lactobacillus gasseri* SBT2055 (LG2055), a bacterial strain derived from a fecal specimen of a healthy adult originally isolated by Fujiwara *et al.* [3] was deposited in the International Patent Organism Depositary, National Institute of Advanced Industrial Science and Technology (Tsukuba, Ibaraki 305–8566, Japan). *Lactobacillus gasseri* SBT0317 (LG0317) was isolated from a dairy product and stocked at Megmilk Snow Brand Co. Ltd. [16]. *Lactobacillus gasseri* JCM1131^T^ (LG1131T) and *Lactobacillus delbrueckii* subspecies (subsp.) *bulgaricus* JCM1002^T^ (LB1002T) were obtained from the Japan Collection of Microorganisms. *Streptococcus thermophilus* ATCC19258^T^ (ST19258T) was obtained from the American Type Culture Collection (Manassas, VA, USA). Each strain was grown at 37°C for 16 h in de Man, Rogosa and Sharpe (MRS) broth (Becton-Dickinson and Company, MD, USA). Harvested cells of each strain were washed twice with saline and once with sterilized water, then lyophilized using a freeze dryer (FDU-2200, Tokyo Rikakikai Co., Ltd., Tokyo, Japan).

#### Measurement of pancreatic lipase activity

Pancreatic lipase activity using fat emulsion as a substrate was determined using the method described by Han *et al.* [17]. A fat emulsion was prepared by sonication of lipid suspensions composed of triolein (80 mg), lecithin (10 mg), and taurocholic acid (5 mg) in 9 ml of TES buffer (0.1 M TES, 0.1 M NaCl, pH 7.0) for 25 min. Bacterial cells of each strain were suspended in TES buffer. For the enzyme reaction, 100 μl of a bacterial cell suspension or orlistat solution and 50 μl of pancreatic lipase (10 units) were added to 100 μl of sonicated substrate suspension (fat emulsion) in a total volume of 250 μl, and the reaction mixture was incubated at 37°C for 30 min. After completion of the reaction, the solution was heated in a boiling water bath for 2 min for enzyme inactivation. The blank of each sample was heated in a boiling water bath for 2 min immediately following addition of the enzyme solution for inactivation. The concentration of released fatty acids was measured using a NEFA C Test Wako. To examine dose-dependency, LG2055 suspensions were prepared at final concentrations ranging from 1–100 μg/ml. The effects of five bacterial strains (LG2055, LG1131T, LG0317, LB1002T and ST19258T) were compared using preparations at final concentrations of 100 μg/ml. Pancreatic lipase activity of each sample was calculated using fatty acid production in the absence of sample as 100%. Lipase activity using 4-MUO as a substrate [18] was determined using the method described by Nakai *et al.* [19]. LG2055 cells were suspended in distilled water. Twenty-five microliters of a LG2055 suspension or an orlistat solution and 50 μl of a 0.1 mM 4-MUO solution dissolved in Tris buffer consisting of 13 mM Tris–HCl, 150 mM NaCl, and 1.3 mM CaCl_2_ (pH 8.0) were mixed in the well of a microtiter plate, and 25 μl of the lipase solution (50 U/ml) in Tris buffer was then added to initiate the enzyme reaction. After incubation at 25°C for 30 min, 100 μl of 0.1 M sodium citrate (pH 4.2) was added to terminate the reaction. The amount of 4-methylumbelliferone released following the lipase reaction was measured using a fluorometrical microplate reader (Varioskan™ Flash, Thermo Fisher Scientific, Inc., MA, USA) at an excitation wavelength of 327 nm and an emission wavelength of 449 nm. The LG2055 suspensions and orlistat solutions were prepared at final concentrations ranging from 1–100 μg/ml, and 0.1–100 μg/ml, respectively. Pancreatic lipase activity of each sample was calculated using 4-methylumbelliferone production in the absence of sample as 100%.

#### Measurement of particle size of fat emulsion

LG2055 was suspended in 1.2 ml of TES buffer and added to 8 ml of the fat emulsion preparation. After addition of LG2055, the pH of the suspension was adjusted to 7.5 using NaHCO_3_, to reflect the *in vivo* condition, in which pancreatic juice containing alkaline sodium (sodium bicarbonate (NaHCO_3_)) is secreted into the small intestine for maintenance of a neutral pH. TES buffer was used as a negative control, whereas a catechin mixture derived from green tea (Wako) was used as a positive control [20]. The prepared final suspensions were incubated at 37°C with constant shaking (100 strokes/min) for 3 h [20]; next, the size distribution and mean fat emulsion droplet sizes were measured using a particle size analyzer (Microtrac ® MT3000II, Nikkiso Co., Ltd, Tokyo, Japan). Suspensions of the five bacterial strains (LG2055, LG1131T, LG0317, LB1002T and ST19258T) were prepared at a final concentration of 100 μg/ml.

### Human study

#### Subjects

Thirty healthy adults (12 men and 18 women) between 27–69 years of age were enrolled in the study. Subjects with a frequency of defecation less than five days per week and severe internal organ disorders, including coronary heart disease, respiratory impairment, endocrinopathy, or alimentary allergy were excluded. None of the subjects consumed special health-promoting foods, took medications known to alter lipid metabolism, or regularly ingested fermented milk.

#### Study design

The study was performed as a double-blind, randomized, placebo-controlled clinical trial, according to the guidelines established in the Declaration of Helsinki. All procedures involving human subjects were approved by the institutional review board of the Miyawaki Orthopedic Clinic (Eniwa City, Hokkaido, Japan) prior to initiation of the study. All subjects provided written informed consent prior to study participation. The study was conducted from January 2014 to February 2014 by a contract research organization, New Drug Research Center, Inc. (Minato-ku, Tokyo, Japan). The clinical trial was registered at the University Hospital Medical Information Network Clinical Trials (No. UMIN000015772).

#### Preparation of the test fermented milk

Two types of fermented milk (FM) were prepared: the active FM containing LG2055 and the control FM lacking LG2055. The active FM was prepared using lactic acid bacteria starter cultures (*Streptococcus thermophilus* and *Lactobacillus delbrueckii* subsp. *bulgaricus*) commonly used for conventional yogurt production and viable LG2055 cells. An FM mixture consisting of approximately 11% skim milk powder with a small amount of flavor, agar, and sucralose as a non-caloric artificial sweetener was inoculated with the yogurt starter cultures and LG2055 cells, then cultured at 40°C for 3.5–4 h. On the initial day, the viable cell count of LG2055 was approximately 5 × 10^9^ CFU/100 g of FM. The control FM was prepared in the same manner, except that LG2055 cells were excluded. These FM preparations were equivalent in energy (146.4 kJ), protein (3.7 g), fat (0.1 g), carbohydrate (4.9 g), and sodium content (40 mg) per 100 g and were indistinguishable in taste. The test FM preparations were kept in cold storage and delivered weekly.

#### Study schedule and protocol

The study length was fourteen days, comprising a seven-day pre-observational period followed by a seven-day FM intake period. Subjects were randomized into the control and active FM groups. To equalize energy and fat intakes, subjects were provided the same diets throughout the entire study period (14 days), consisting of different menus at each meal, each day of the study. Energy intake was set at approximately 8368 and 9623.2 kJ per day in women and men, respectively; the amount of fat intake was set at approximately 70 and 85 g per day (31.5 and 33.3 percent energy) in women and men, respectively. After the seven-day pre-observational period, both groups started consuming the test FM for seven days. The subjects consumed FM at 100 g/d with meals (at either breakfast, lunch or dinner) and they were asked to maintain their normal lifestyle habits, including exercise routines. All subjects maintained a detailed dietary record during the entire study period. Fecal samples were collected during the final three days of the pre-observational (days 5–7) and FM intake periods (days 12–14). Fecal samples were weighed and immediately frozen for storage. Body weight, body fat percentage, blood pressure, pulse rate, and fasting blood parameters (triacylglycerol, total cholesterol, high density lipoprotein cholesterol, NEFA, glucose, total protein, aspartate aminotransferase, alanine aminotransferase, alkaline phosphatase, and gamma-glutamyl transpeptidase) were determined at the beginning of the test period (day 1) and the day after the end of the experimental period (day 15). Physician interviews were also performed at each time point (day 1 and 15). Information regarding subjective symptoms such as headache, nausea, and abdominal pain were obtained through a physician interview at each time point (day 1 and 15).

#### Measurement of fecal fat concentration

Fecal fat level was determined according to the methods described by van de Kamer *et al.* [21].

#### Physical characteristics and blood sample analyses

Blood analyses were performed by SRL, Inc. (Shinjuku-ku, Tokyo, Japan). The blood samples were centrifuged at 188 × *g* for 10 min at 4°C, and the supernatant was stored at a temperature below −30°C until analysis. The concentration of serum NEFA was measured using a biochemical autoanalyzer JCA-BM6010 (JEOL Ltd., Tokyo, Japan). The concentrations of other serum parameters (triacylglycerol, total cholesterol, high density lipoprotein cholesterol, glucose, total protein, aspartate aminotransferase, alanine aminotransferase, alkaline phosphatase, and gamma-glutamyl transpeptidase) were measured using a biochemical autoanalyzer AY5400 (Beckman Coulter Inc., CA, USA).

#### Statistical analysis

All *in vitro* experiments were performed three times and data were expressed as means with standard deviation (SD). The Tukey-Kramer post-hoc test was used for multiple comparisons between strains. In the human study, the differences between the pre- and post-FM intake periods, and the differences between the control and active FM groups, were evaluated using Student’s paired *t*-test and Student’s unpaired *t*-test, respectively. A *P* value < 0.05 was considered statistically significant.

## Results

### Effects of LG2055 on pancreatic lipase activity *in vitro*

**Figure 1:**
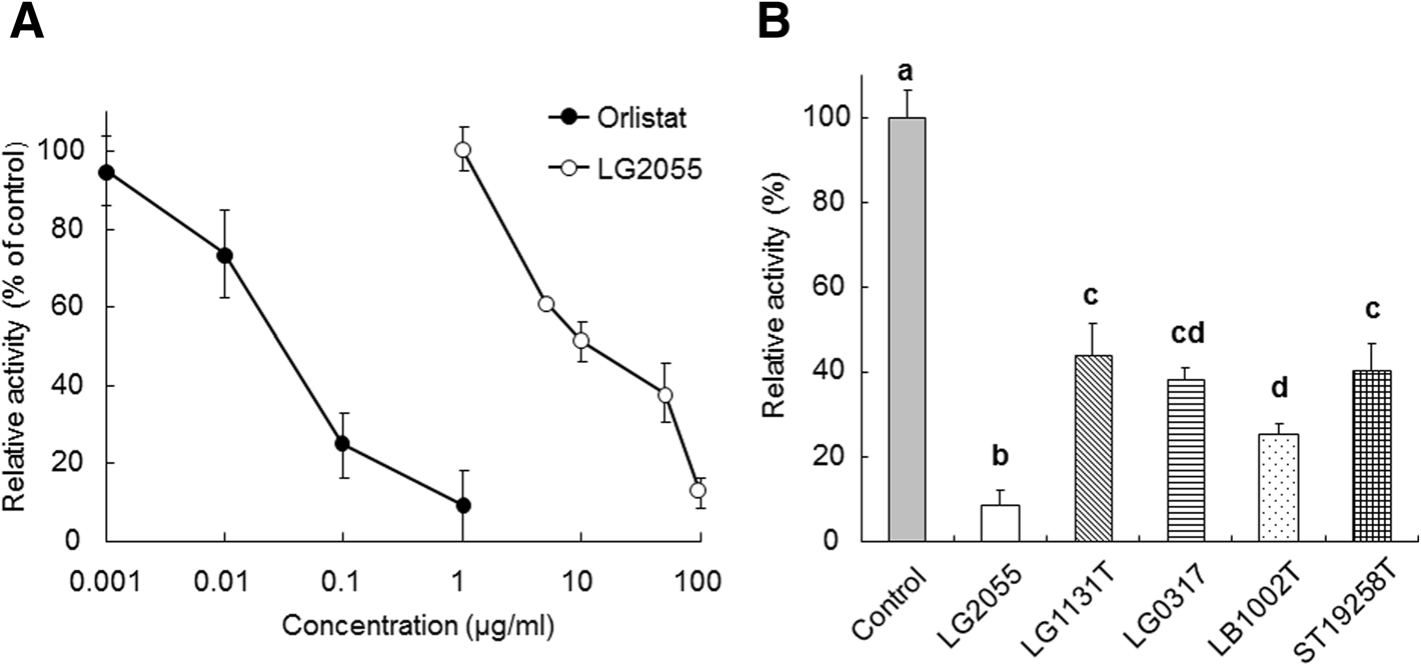
**Suppressive effect of lactic acid bacteria on pancreatic lipase-mediated hydrolysis of triolein in an emulsion.** Substrate suspensions (fat emulsion) were incubated with pancreatic lipase (200 U/ml) and lactic acid bacterial cells or orlistat for 30 min at 37°C. After boiling for 2 min, released fatty acids were quantified. The suppressive activity was calculated for fatty acid production, in which the activity in the absence of sample was represented as 100%. **(A)** *Lactobacillus gasseri* SBT2055 (LG2055) suppressed the release of fatty acids from fat emulsion in a dose-dependent manner. **(B)** Comparison of the capacity of various lactic acid bacteria in suppressing fatty acid release. Each of the five bacterial strains (LG2055, *Lactobacillus gasseri* SBT0317 (LG0317), *Lactobacillus gasseri* JCM1131^T^ (LG1131T), *Lactobacillus delbrueckii* subsp. *bulgaricus* JCM1002^T^ (LB1002T), and *Streptococcus thermophilus* ATCC19258^T^ (ST19258T)) was added to the lipase reaction solution at 100 μg/ml. Values are means with standard deviations for triplicate experiments. Statistical differences between the strains were analyzed using the Tukey-Kramer post-hoc test and significant differences (*P* < 0.05) are indicated using different letters.

We first examined the effect of LG2055 on pancreatic lipase activity using a fat emulsion as a substrate *in vitro* (Figure 1). The lipase inhibitor orlistat [13] strongly inhibited lipase activity in a dose-dependent manner (0.001–1 μg/ml); LG2055 also suppressed lipase activity in a dose-dependent manner (1–100 μg/ml) (Figure 1A). All the examined lactic acid bacterial species and strains significantly suppressed lipase activity at a final concentration of 100 μg/ml compared with the control lacking bacteria. Furthermore, LG2055 strongly suppressed lipase activity compared with the other four strains (LG1131T, LG0317, LB1002T, and ST19258T) (Figure 1B).

**Figure 2:**
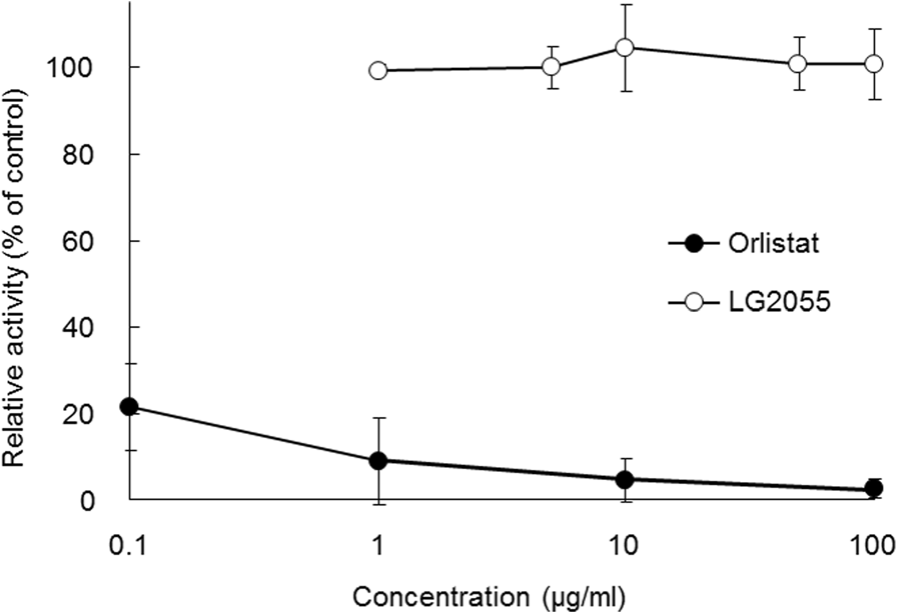
**Effect of** ***Lactobacillus gasseri*** **SBT2055 (LG2055) on pancreatic lipase activity using 4-methylumbelliferyl oleate (4-MUO) as substrate.** Various concentrations of LG2055 and orlistat, and 0.1 mM 4-MUO solution were mixed in the well of a microtiter plate, and the lipase solution (50 U/ml) was added. After incubation at 25°C for 30 min, 0.1 M sodium citrate (pH 4.2) was added to terminate the reaction. The suppressive activity was calculated based on the amount of 4-methylumbelliferone, in which the activity in the absence of sample was represented as 100%. Values are means with standard deviations for triplicate experiments.

When lipase activity was measured using 4-MUO as a substrate (Figure 2), orlistat was strongly inhibitory, whereas LG2055 had no effect at concentrations ranging from 1–100 μg/ml. Thus, LG2055 did not suppress lipase activity using 4-MUO as a substrate. The other four strains (LG1131T, LG0317, LB1002T, and ST19258T) did not also suppress the lipase activity using 4-MUO (data not shown).

### Effects of LG2055 on fat emulsion droplet size *in vitro*

The average fat emulsion droplet size remained mostly constant following incubation for 3 h in the absence of LG2055 (shortly after preparation: 2.00 ± 0.41 μm, after incubation: 2.21 ± 0.87 μm), indicating that the fat emulsion preparation was stable for a minimum of 3 h.

**Figure 3:**
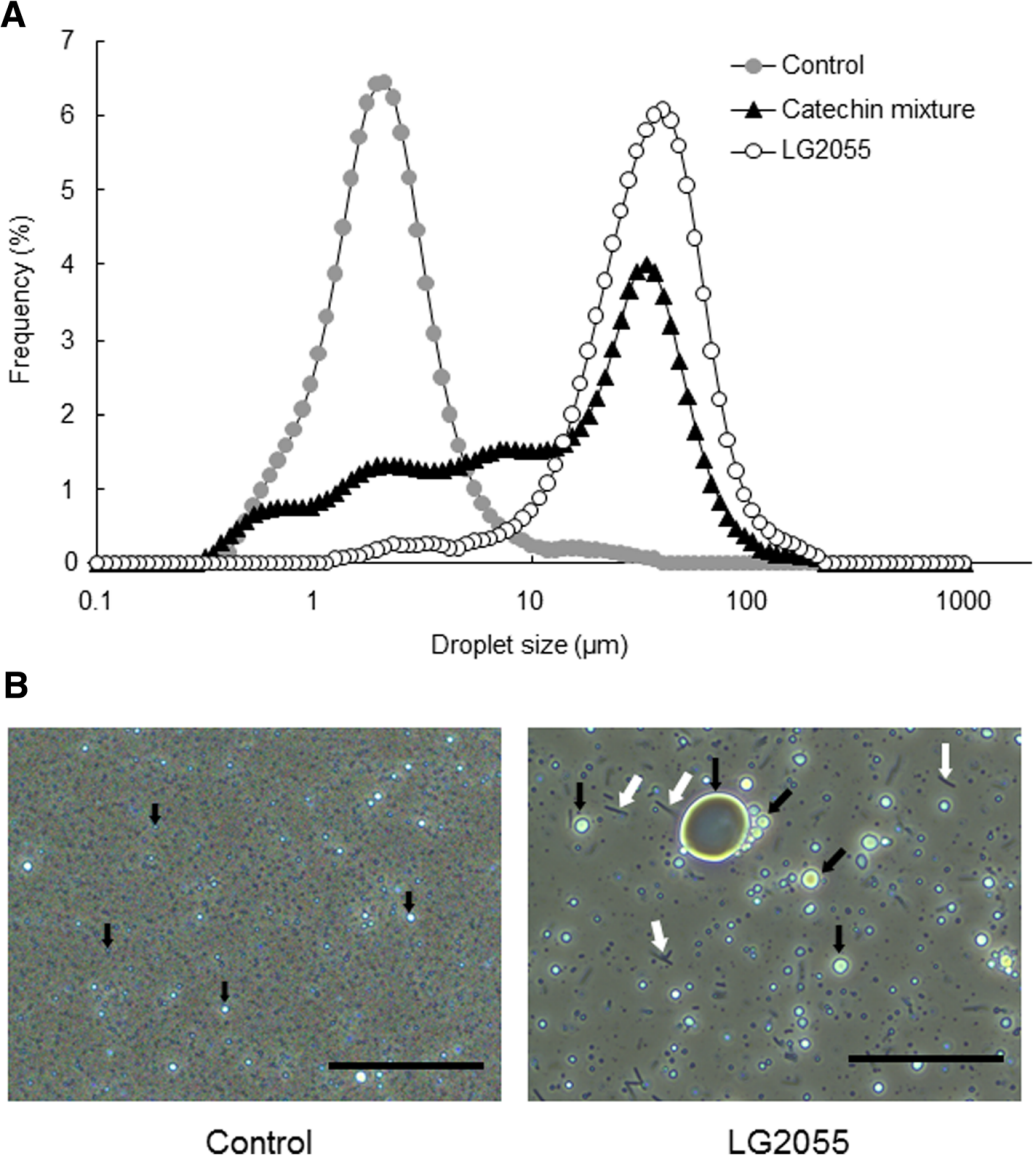
**Effect of** ***Lactobacillus gasseri*** **SBT2055 (LG2055) on fat emulsion droplet size** ***in vitro.*** LG2055 or a catechin mixture was added to the fat emulsion preparation. The suspension was incubated at 37°C with constant shaking for 3 h and the size distribution of fat emulsion was measured using a particle size analyzer. No sample (only TES buffer) was added to the negative control and the catechin mixture was used as a positive control. **(A)** Changes in emulsion droplet size distribution after incubation with 1000 μg/ml of LG2055 or catechin mixture. The distributions are an average of three determinations. **(B)** Phase-contrast micrographs (scale bar: 50 μm). Black and white arrows indicate the representative fat emulsions and LG2055 cells, respectively.

The catechin mixture (1000 μg/ml) increased fat emulsion droplet size, as previously reported [20]. LG2055 significantly modified droplet size distribution at the same concentration as the catechin mixture (Figure 3A). Enlargement of the fat emulsion by LG2055 was also observed in phase-contrast micrographs (Figure 3B).

**Figure 4:**
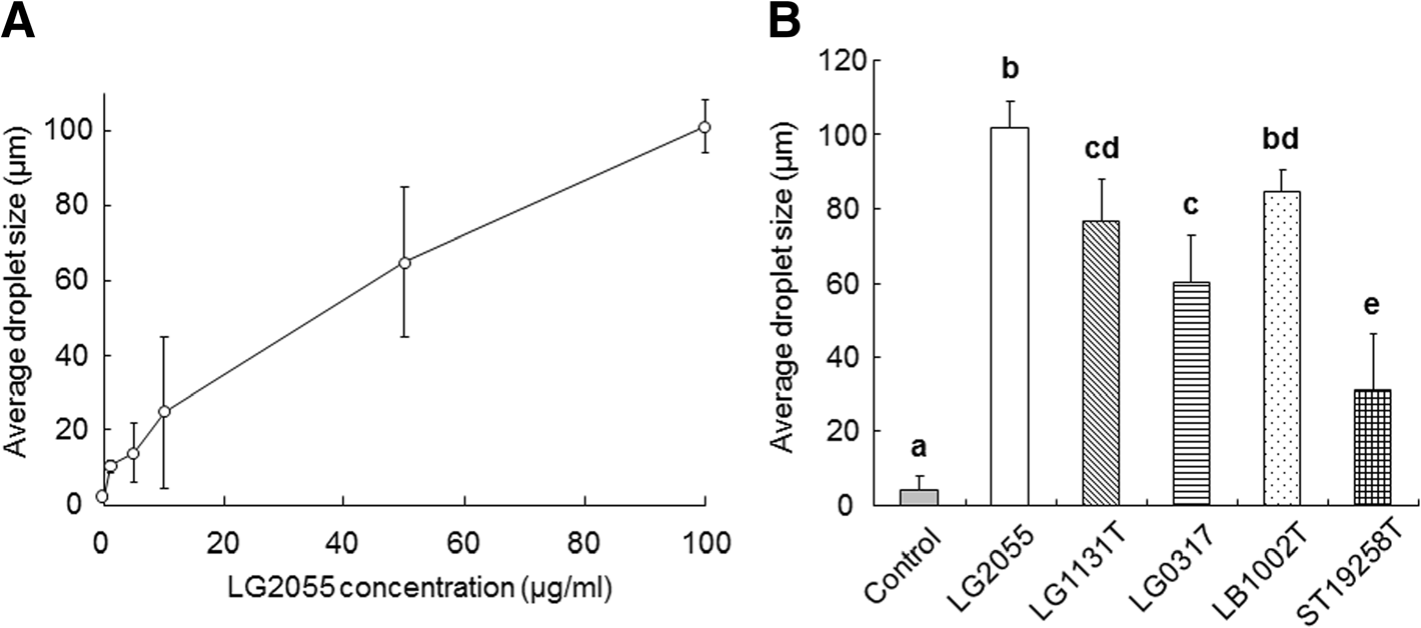
**Increased effects of** ***Lactobacillus gasseri*** **SBT2055 (LG2055) on fat emulsion droplet size. (A)** The dose-dependency of LG2055 on the average fat emulsion droplet size. **(B)** Comparison among lactic acid bacteria at 100 μg/ml. Fat emulsion droplet size was measured in the same manner as described in Figure 3. Values are means with standard deviations for triplicate experiments. Statistical differences between the strains were analyzed using the Tukey-Kramer post-hoc test and significant differences (*P* < 0.05) are indicated using different letters.

LG2055 dose-dependently increased the average fat emulsion droplet size at concentrations ranging from 1–100 μg/ml (Figure 4A). All five strains of lactic acid bacteria (100 μg/ml) significantly increased the average fat emulsion droplet size compared with the control lacking bacteria. In addition, LG2055 significantly increased droplet size compared with the other strains (Figure 4B).

### Clinical study of fecal fat excretion

The physical and biochemical blood characteristics of the subjects were not significantly different between groups (Table 1). No subject dropped out during the study.

**Table 1:** **Baseline characteristics of the study subjects**

**Parameters**	**Control**	**Active**
Number of subjects	15	15
Gender		
Male	6	6
Female	9	9
Age (years)	40.1 ± 9.5	42.6 ± 10.8
Height (cm)	162.2 ± 6.4	163.6 ± 9.4
Body weight (kg)	57.3 ± 6.4	58.8 ± 9.6
Body fat percentage (%)	24.9 ± 6.0	25.4 ± 6.4
BMI (kg/m^2^)	21.8 ± 2.5	21.9 ± 2.8
Systolic blood pressure (mm Hg)	116.6 ± 13.4	120.9 ± 15.5
Diastolic blood pressure (mm Hg)	69.0 ± 9.4	77.0 ± 13.0
Pulse rate (beats/min)	67.2 ± 10.3	69.1 ± 7.3
Triacylglycerol (mg/dl)	65.1 ± 22.0	68.0 ± 30.1
Total cholesterol (mg/dl)	197.1 ± 24.7	203.7 ± 31.0
Non-esterified fatty acid (μEq/l)	398.5 ± 169.5	393.3 ± 173.7
Glucose (mg/dl)	82.4 ± 6.6	86.3 ± 5.0

Values are means ± SD.

The effects of the control and active FM preparations on physical and biochemical blood parameters are listed in Table 2. Body weight, body mass index (BMI), and total cholesterol levels significantly decreased similarly in both groups; alanine aminotransferase (ALT) and gamma-glutamyl transpeptidase (γ-GTP) significantly decreased in the control FM group, and pulse rate significantly decreased in the active FM group but remained within the normal range. Based on daily records and physician interviews, no irregularities in daily life or adverse events related to FM consumption were observed during the study period, (data not shown).

**Table 2:** **Initial (day 1) and final (day 15) values of physical characteristics and biochemical blood parameters (n = 15)**

**Parameters**	**Control**		**Active**	
	**day 1**	**day 15**	**day 1**	**day 15**
Body weight (kg)	57.4 ± 6.5	55.8 ± 6.0**	58.4 ± 9.4	57.2 ± 9.2**
Body fat percentage (%)	24.2 ± 6.4	24.2 ± 5.6	25.1 ± 6.6	25.1 ± 6.9
BMI (kg/m^2^)	21.9 ± 2.6	21.3 ± 2.4**	21.8 ± 2.8	21.4 ± 2.9**
Systolic blood pressure (mm Hg)	117.3 ± 15.5	117.9 ± 13.6	115.9 ± 14.4	118.6 ± 15.2
Diastolic blood pressure (mm Hg)	68.7 ± 10.1	69.1 ± 10.4	71.9 ± 11.2	73.0 ± 11.6
Pulse rate (beats/min)	67.0 ± 5.9	65.9 ± 10.6	71.9 ± 9.0	67.0 ± 8.3*
Triacylglycerol (mg/dl)	67.5 ± 33.5	59.5 ± 21.3	67.2 ± 37.2	66.5 ± 32.3
Total cholesterol (mg/dl)	188.5 ± 17.9	176.1 ± 27.4*	192.7 ± 35.4	179.7 ± 28.0*
HDL cholesterol (mg/dl)	68.1 ± 10.3	68.7 ± 11.1	66.5 ± 11.8	65.6 ± 11.5
Non-esterified fatty acid (μEq/l)	331.8 ± 105.5	372.1 ± 135.1	413.1 ± 223.8	462.9 ± 169.2
Glucose (mg/dl)	85.7 ± 10.0	82.5 ± 5.8	85.3 ± 6.0	84.0 ± 6.2
Total protein (g/dl)	7.4 ± 0.3	7.4 ± 0.5	7.5 ± 0.4	7.5 ± 0.4
AST (U/l)	19.8 ± 4.6	19.1 ± 4.1	19.4 ± 4.5	19.1 ± 4.6
ALT (U/l)	20.3 ± 11.6	18.2 ± 9.4*	15.7 ± 5.6	14.3 ± 5.0
ALP (U/l)	185.4 ± 35.1	176.2 ± 35.7	185.1 ± 38.6	188.3 ± 30.4
γ-GTP (U/l)	23.2 ± 14.1	18.6 ± 9.1*	21.3 ± 10.5	18.5 ± 8.8

*Abbreviations*: *HDL* high density lipoprotein, *AST* aspartate aminotransferase, *ALT* alanine aminotransferase, *ALP* alkaline phosphatase, *γ-GTP* gamma-glutamyl transpeptidase.

Values are means ± SD.

Significantly different from the initial values within the group; *P < 0.05, **P < 0.01.

**Figure 5:**
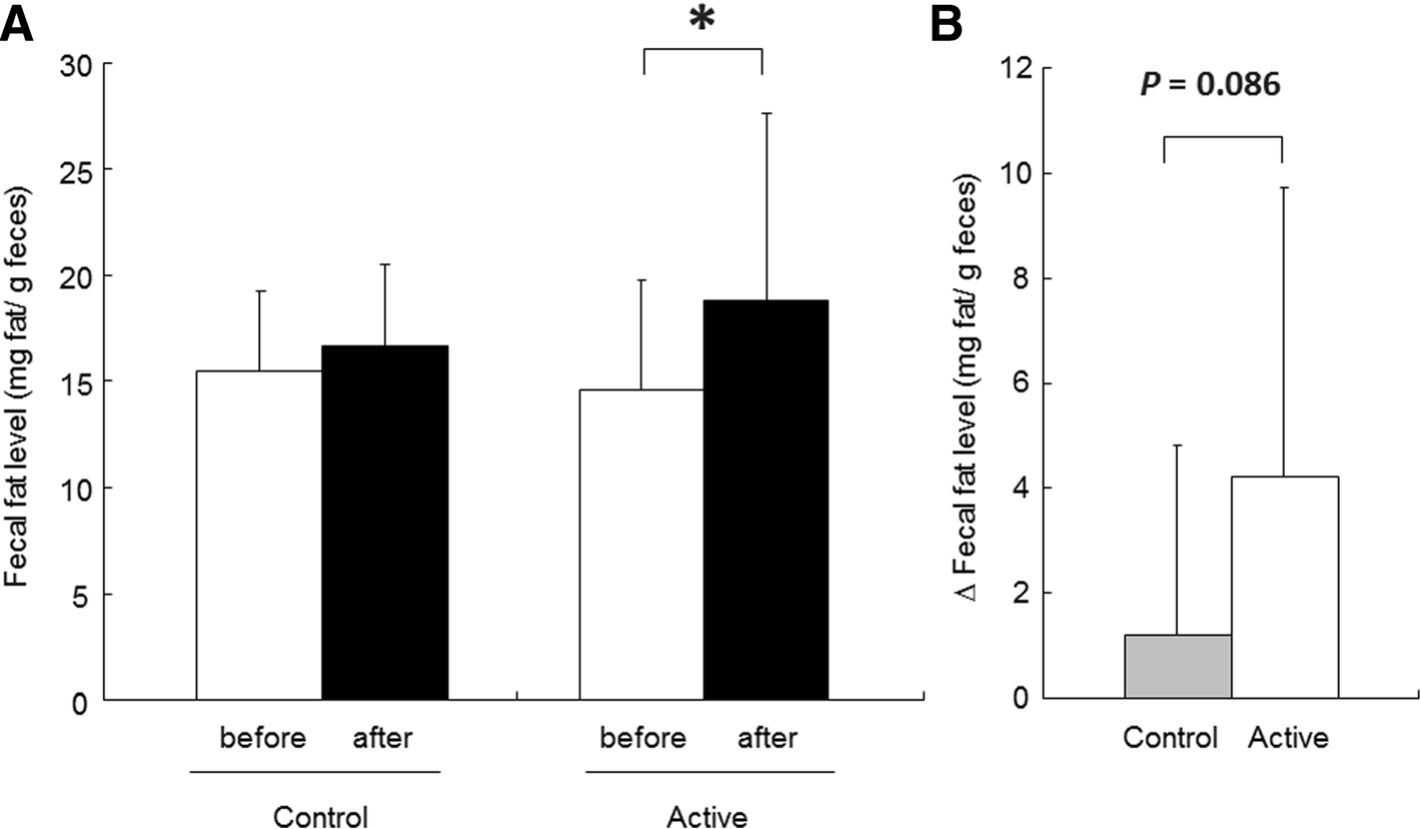
**Effect of intake of FM containing** ***Lactobacillus gasseri*** **SBT2055 (LG2055) on fecal fat excretion in humans. (A)** Fecal samples were collected during the final three days of the pre-observational period (before) and FM intake period (after), and fecal fat levels were determined. **(B)** The amount of change in the fecal fat levels in the control and active FM groups. Values were calculated by subtracting fecal fat levels at pre-observational period from those at FM intake period. Values are means with standard deviations. Statistical analysis was performed using paired Student’s *t*-test **(A)** and unpaired Student’s *t*-test **(B)**. An asterisk indicates a significant difference (*P* < 0.05).

There were no statistically significant differences in wet weight of feces between groups (270.1 ± 159.0 g in control FM group vs. 336.8 ± 116.9 g in active FM group before FM intake; 264.9 ± 151.0 g in control FM group vs. 304.4 ± 128.7 g in active FM group after FM intake). After FM intake, fecal fat levels were significantly increased compared with those observed before FM intake in the active FM group, whereas no increase was observed in the control FM group (Figure 5A). The amount of change between pre- and post-FM intake periods increased in the active FM group compared with the control FM group, although the difference was not statistically significant (*P* = 0.086).

## Discussion

The suppression of pancreatic lipase-mediated fat hydrolysis is an effective strategy for prevention of obesity and hyperlipidemia. Pancreatic lipase inhibitors such as orlistat are currently used clinically as pharmaceutical agents [22]. In addition, it has been reported that dietary compounds, including chitosan [17], saponin [23], and oolong tea polyphenols [19], inhibit pancreatic lipase, consequently suppressing dietary lipid absorption. In this study, we observed that LG2055, a probiotic bacterium displaying anti-obesity properties, suppressed fatty acid release from fat emulsion *in vitro* (Figure 1A). Moreover, it was reported that LG2055 suppresses lipid absorption in the small intestine *in vivo* [4]. Taken together, these findings suggest that suppression of pancreatic lipase-mediated fat hydrolysis could represent a mechanism by which LG2055 mediates suppression of lipid absorption. Consistent with our findings, Matsumura [24] and Zhou *et al.* [25] reported that certain strains of *Lactobacillus* inhibit pancreatic lipase. The present study further demonstrated that all five examined lactic acid bacterial strains, including LG2055, had an ability to suppress lipase activity (fatty acid release from fat emulsion) to a certain extent (Figure 1B); however, LG2055 significantly suppressed fatty acid release from fat emulsion compared with the other strains, indicating a more potent effect. These results suggest that many strains of lactic acid bacteria likely have the ability to suppress lipase activity. However, LG2055 could represent a specific strain with an increased potential for lipase suppression among lactic acid bacteria.

The suppression of fat hydrolysis is mainly classified into two types: the first is suppression mediated by direct interaction between the inhibitor and enzyme, whereas the second is associated with modification of fat emulsion properties [26,27]. If LG2055 directly inhibits lipase-mediated hydrolysis, its suppressive effect should be observed when using a synthetic substrate such as 4-MUO. We observed that orlistat inhibited lipase activity using 4-MUO as a substrate, consistent with results using fat emulsion. Conversely, LG2055 was not inhibitory (Figure 2), indicating that LG2055 does not directly inhibit pancreatic lipase. We also confirmed that all four tested strains (LG1131T, LG0317, LB1002T, and ST19258T) did not inhibit lipase activity using 4-MUO as a substrate (data not shown). These results imply that LG2055 acts on the fat emulsion of a substrate rather than directly on the enzyme, leading to suppression of lipase-mediated hydrolysis. Fat emulsion interface properties, namely droplet size and specific surface area, control lipase-mediated fat emulsion hydrolysis [14,28,29]. Therefore, we examined whether LG2055 modifies fat emulsion properties by measuring fat emulsion droplet size upon mixing with LG2055. LG2055 promoted fat emulsion enlargement (Figure 3B) and dose-dependently increased fat emulsion droplet size (Figure 4A). An increase in droplet size is known to be associated with a decrease in specific surface area, resulting in delayed lipase-mediated hydrolysis. It has been reported that green tea extract [15], catechin mixture [20], and green coffee bean extract [20] increase fat emulsion droplet size, resulting in suppression of lipid absorption. However, there are no reports evaluating the effect of lactic acid bacteria on fat emulsion droplet size. Matsumura [24] and Zhou *et al*. [25] demonstrated that certain *Lactobacillus* strains inhibited pancreatic lipase activity but did not examine the mechanisms involved. To our knowledge, this is the first report describing an enlargement of fat emulsion by probiotics. With the exception of LG2055, the four tested strains also promoted an increase in fat emulsion droplet size (Figure 4B). However, LG2055 significantly increased droplet size compared with the other strains. These results are consistent with the strong suppressive effects of LG2055 on lipase-mediated fat hydrolysis.

It remains unclear how LG2055 increases fat emulsion droplet size. One possibility is it that an interaction between LG2055 and bile acids (e.g. taurocholic acid) could contribute to the modification of fat emulsion properties. Interestingly, it was reported that certain *Lactobacillus* strains have an ability to bind and deconjugate taurocholic acid [30]. LG2055 also has an ability to deconjugate taurocholic acid [16]. Bile acids have amphipathic properties and are critical for the emulsification of dietary lipids in the intestine [31]. As our preliminary study demonstrated, LG2055 did not increase fat emulsion droplet size without inclusion of taurocholic acid prepared by the method reported by Shishikura *et al.* [15] (data not shown). LG2055 potentially interacts with bile acids and destabilizes the fat emulsion, resulting in its coalescence. Usman *et al.* previously reported that LG0317, a strain included in the present study, had a decreased capacity to deconjugate taurocholic acid compared with LG2055 [16]. In our study, LG0317 had a decreased effect on fat emulsion enlargement. These findings indicate the importance of the interaction between taurocholic acid and lactic acid bacteria for promotion of increased fat emulsion droplet size.

We recently reported that consumption of FM containing LG2055 decreased postprandial serum triacylglycerol and non-esterified fatty acid (NEFA) concentrations in peripheral blood after intake of oral fat-loading test meals compared with consumption of FM without LG2055 [10]. Although triacylglycerol and NEFA concentrations in peripheral blood are important biomarkers of fat absorption, they are affected by metabolism in organs such as the liver [32]. Thus, we evaluated fecal lipid excretion to assess more directly the effect of LG2055 on lipid absorption in humans. Our data show that consumption of FM containing LG2055 increased fecal fat excretion (Figure 5A/B). Given that LG2055 suppressed pancreatic lipase-mediated fat hydrolysis *in vitro*, its effects on fecal fat excretion are likely associated with suppression of lipase-mediated fat hydrolysis, resulting in decreased lipid absorption. In this study, we observed that BMI and total serum cholesterol concentration were significantly decreased, but remained within the normal range, after FM intake in both the control and active FM groups (Table 2). We consider that this is because energy intake by the subjects was moderately controlled, or beneficial effects of fermented milk on lipid metabolism [33] were taken. Consumption of LG2055 for seven days did not significantly influence body fat percentage. However, constant ingestion of LG2055 over a prolonged period could reveal more pronounced anti-obesity effects, as we previously demonstrated when LG2055 was consumed daily for 12 weeks [9].

## Conclusions

In conclusion, our findings clearly demonstrate that LG2055 modifies the physicochemical properties of fat emulsion by increasing droplet size and suppressing fatty acid release from fat emulsion. These data provide a mechanism by which LG2055 mediates the suppression of lipid absorption and an increase in fecal fat excretion in humans.

## Abbreviations

LG2055: *Lactobacillus gasseri* SBT2055
BMI: Body mass index
4-MUO: 4-methylumbelliferyl oleate
TES: *N*-tris (hydroxymethyl) methyl-2-aminoethanesulfonic acid
LG0317: *Lactobacillus gasseri* SBT0317
LG1131T: *Lactobacillus gasseri* JCM1131^T^
LB1002T: *Lactobacillus delbrueckii* subsp. *bulgaricus* JCM1002^T^
ST19258T: *Streptococcus thermophilus* ATCC19258^T^
FM: Fermented milk
NEFA: Non-esterified fatty acid
